# Multimodal photoacoustic microscopy, optical coherence tomography, and fluorescence imaging of USH2A knockout rabbits

**DOI:** 10.1038/s41598-023-48872-1

**Published:** 2023-12-12

**Authors:** Van Phuc Nguyen, Justin Hu, Josh Zhe, Eugene Y. Chen, Dongshan Yang, Yannis M. Paulus

**Affiliations:** 1 Department of Ophthalmology and Visual Sciences, University of Michigan, 1000 Wall Street, Ann Arbor, MI 48105 USA; 2 Department of Biomedical Engineering, University of Michigan, Ann Arbor, MI 48105 USA; 3 Department of Internal Medicine, Center for Advanced Models for Translational Sciences and Therapeutics, University of Michigan, 2800 Plymouth Rd NCRC B26-355S, Ann Arbor, MI 48109-2800 USA

**Keywords:** Biomedical engineering, Retinal diseases

## Abstract

Usher syndrome type 2A (USH2A) is a genetic disorder characterized by retinal degeneration and hearing loss. To better understand the pathogenesis and progression of this syndrome, animal models such as USH2A knockout (USH2AKO) rabbits have been developed. In this study, we employed multimodal imaging techniques, including photoacoustic microscopy (PAM), optical coherence tomography (OCT), fundus autofluorescence (FAF), fluorescein angiography (FA), and indocyanine green angiography (ICGA) imaging to evaluate the retinal changes in the USH2AKO rabbit model. Twelve New Zealand White rabbits including USH2AKO and wild type (WT) were used for the experiments. Multimodal imaging was implemented at different time points over a period of 12 months to visualize the progression of retinal changes in USH2AKO rabbits. The results demonstrate that ellipsoid zone (EZ) disruption and degeneration, key features of Usher syndrome, began at the age of 4 months old and persisted up to 12 months. The EZ degeneration areas were clearly observed on the FAF and OCT images. The FAF images revealed retinal pigment epithelium (RPE) degeneration, confirming the presence of the disease phenotype in the USH2AKO rabbits. In addition, PAM images provided high-resolution and high image contrast of the optic nerve and the retinal microvasculature, including retinal vessels, choroidal vessels, and capillaries in three-dimensions. The quantification of EZ fluorescent intensity using FAF and EZ thickness using OCT provided comprehensive quantitative data on the progression of degenerative changes over time. This multimodal imaging approach allowed for a comprehensive and non-invasive assessment of retinal structure, microvasculature, and degenerative changes in the USH2AKO rabbit model. The combination of PAM, OCT, and fluorescent imaging facilitated longitudinal monitoring of disease progression and provided valuable insights into the pathophysiology of USH2A syndrome. These findings contribute to the understanding of USH2A syndrome and may have implications for the development of diagnostic and therapeutic strategies for affected individuals. The multimodal imaging techniques employed in this study offer a promising platform for preclinical evaluation of potential treatments and may pave the way for future clinical applications in patients with Usher syndrome.

## Introduction

Usher syndrome (USH) is an autosomal recessive genetic disorder characterized by sensorineural hearing loss (SNHL), type-dependent vestibular impairment, and retinitis pigmentosa (RP), an inherited degenerative eye disease that progressively causes severe vision loss^1^. In RP, the photoreceptor cells deteriorate and lose function over time, resulting in night blindness and peripheral vision loss before progressing to permanent, late central vision loss. USH is the most prevalent condition of combined vision and hearing impairment, with an estimated over 400,000 people impacted globally^2^_._ USH accounts for approximately 50% of total hereditary deaf-blindness cases^3^. USH can be categorized into three subtypes (I, II, and III), with each subtype decreasing in condition severity, starting with Type I. Each subtype varies in age of onset of SNHL and RP as well as in whether vestibular dysfunction is present. Type II (USH2) is the most common USH subtype, making up nearly half of all USH cases^4^. Individuals with USH2 experience moderate to severe SNHL at birth followed by RP onset in adolescence or early adulthood but maintain normal vestibular function. Currently, 16 USH causative genes have been identified and confirmed, with three genes, USH2A, GPR98 (USH2C), and DFNB31 (USH2D), corresponding to USH2. USH2A mutations are the most common, making up about 85% of all USH2 cases^5^. Although numerous clinical and non-clinical studies have investigated USH, there is currently no cure for the condition. In addition to early screening and diagnosis of USH, hearing aids and cochlear implants can help manage hearing loss. However, there are far less effective management resources for RP and no treatment options, leaving a significant need for research to identify therapies to address progressive vision loss and eventual blindness stemming from USH.

Animal models of USH2A have been previously developed in mice and zebrafish^6–10^. Although mice share many anatomical similarities of the eye with humans, the mouse model has multiple limitations that hinder the translation of results to clinical applications and treatment development. One is that the average mouse eyeball is much smaller in size—nearly 8 times smaller in diameter—than the human eye^11,12^. However, the most prominent limitation is the significant phenotypic difference between human patients and current USH2A mutant mouse models^13^. This discrepancy is problematic, as the retinal degeneration phenotype is typically very weak or absent among USH2A knockout mouse models. Zebrafish models are promising in that they are being increasingly used for ophthalmic research. There are several USH2A mutant zebrafish models that exhibit early onset retinal degeneration as well as a recent model that displays slower, progressive retinal degeneration^14^. However, there are notable anatomical differences between zebrafish and human eyes, including variation in retinal structure and photoreceptor regeneration capacity that may pose problems in translational studies. Accordingly, the development of a larger mammalian model that more closely resembles the anatomy, physiology, and RP pathogenesis of the human eye may provide greater insight into USH2A mutation-induced RP and stimulate the advancement of clinical therapies and interventions.

There are very few studies investigating USH2A that involve a larger mammalian model. Recently, our group generated a successful USH2A knockout (USH2AKO) model in New Zealand White rabbits that shares resemblance with human USH2A progression and holds promise for future USH2A research^15^. To better understand the microvasculature changes that occur over time, additional imaging modalities like photoacoustic microscopy (PAM) have recently been developed that may provide greater insight and characterization of the USH2AKO rabbit model. Photoacoustic microscopy (PAM) is a valuable imaging technique used in animal models for ocular research^16–20^. PAM relies on the photoacoustic effect, where tissue absorbs pulsed laser light and emits ultrasound waves as a result. These ultrasound waves are detected and converted into images, allowing researchers to visualize various structures within the eye. PAM offers high-resolution imaging capabilities, enabling the visualization of ocular structures such as retinal layers, blood vessels, and the choroid in great detail^21–23^. PAM is particularly useful in animal models of ocular diseases such as glaucoma, age-related macular degeneration (AMD), diabetic retinopathy, and retinal vascular disorders^24,25^. PAM can be used to track disease progression and assess the effectiveness of potential treatments. Another benefit of PAM is its ability to integrate with other optical systems to establish a multimodal imaging system. Song and Jao et al*.* have developed a PAM and OCT system to image the retina, melanin, and CNV in mice^26,27^. A limitation of this study lies in the fact that it can only visualize the structure of mouse eyes, which are considerably smaller than human eyes. The size of a mouse eyeball is roughly estimated to be eight times smaller than that of a human eye^11,28^. This constraint may hinder the translation of this system for clinical applications.

To overcome such challenges, our group has developed a multimodal PAM, OCT, and fluorescence microscopy (FM) imaging system for application in the eyes of large animals like rabbits^29–40^. We applied our technique to visualize different ocular pathologies including retinal vein occlusion (RVO)^34,39,41^, choroidal vascular occlusion^42^, choroidal neovascularization (CNV)^29,30,32,43–46^, retinal neovascularization (RNV)^47^, and corneal neovascularization^19,48^. The uniqueness of our system relies on the low excitation laser energy (~ 80 nJ), which is about half below the ANSI safety limit (~ 160 nJ at 578 nm)^36^. Our novel multimodal imaging system can provide a high aerial spatial resolution of 4.1 μm and 3.8 μm for PAM and OCT, respectively, as described previously^36^. The acquisition time is 65 s to achieve three-dimensional imaging with a scanning resolution of 256 × 256 pixels. By employing the advantages of the high-resolution multimodal PAM, OCT, and FA imaging system, this study aims to observe and track the progression of USH2A in the rabbit model. This imaging system can provide detailed information about the structure and function of the retina and other ocular tissues, allowing researchers to monitor changes or abnormalities associated with USH2A over time. The combination of PAM, OCT, and FM in this study offers complementary imaging modalities that can provide deeper insights into the disease progression and potentially contribute to the development of diagnostic and therapeutic approaches for USH2A and related disorders.

## Results

### In vivo OCT imaging of the USH2AKO rabbit model

**Figure 1:**
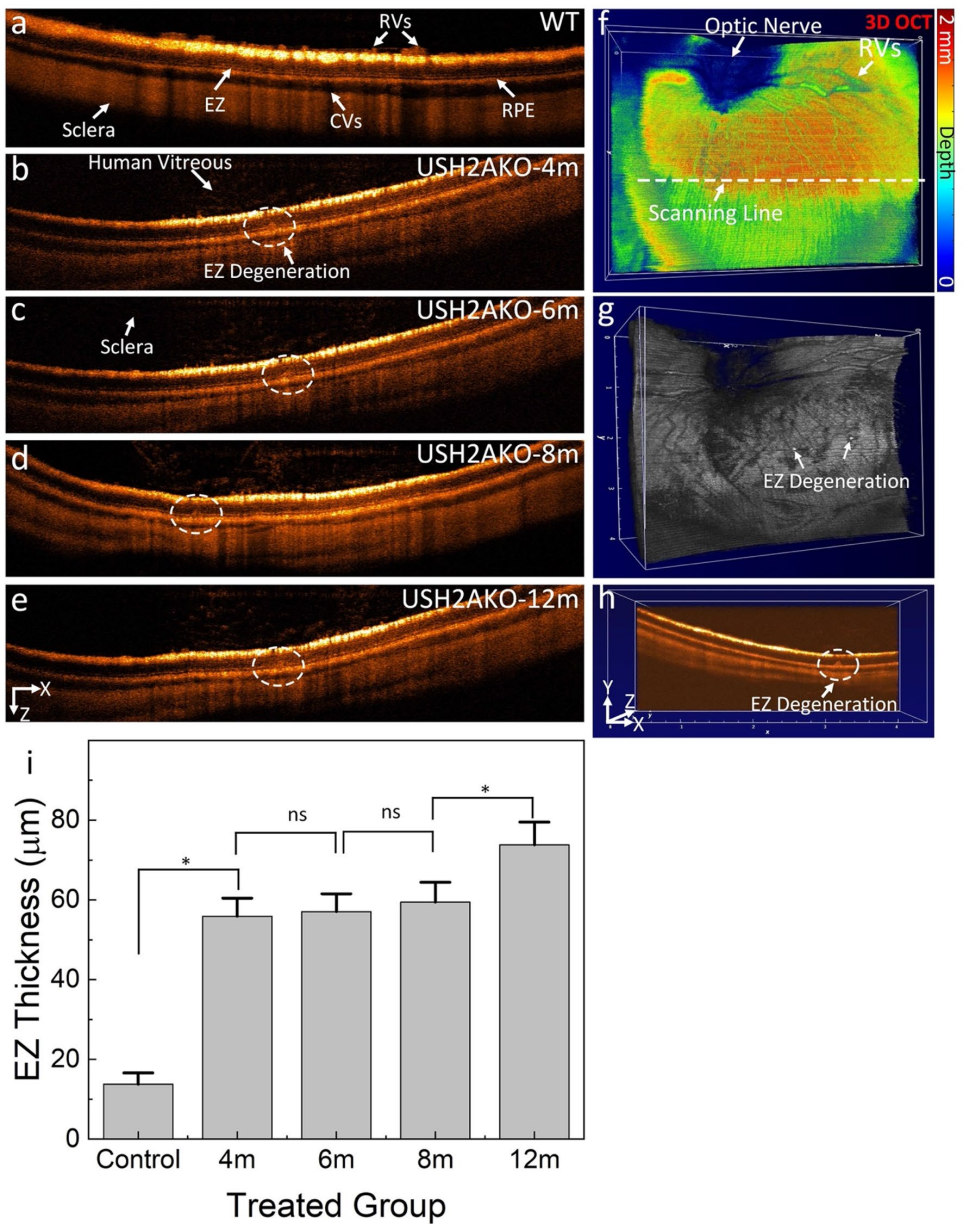
Longitudinal 2D OCT imaging reveals progressive ellipsoid zone (EZ) degeneration in USH2AKO rabbits: **(a**) Control image showing different retinal layers including RVs, CVs, EZ, RPE, and sclera. (**b**–**e**) Images at 4–12 months old, demonstrating persistent EZ degeneration. Noteworthy is persistence and progression of EZ degeneration from 4 to 12 months old. (**f**) Enhanced 3D OCT volumetric visualization at 12 months reveals optic nerve and RVs location. (**g**) Orthogonal images from a single B-Scan illustrate the detected EZ area (white dotted circle). (**h**) *En face* 3D OCT emphasizes the surface of the EZ region. (**i**) A graph depicting the quantification of EZ thickness, illustrating the progression of EZ degeneration over time. The data is presented as the mean ± standard deviation (n = 3, *p* < 0.005).

High-resolution spectral domain optical coherence tomography (SD-OCT) imaging was employed to visualize the progression of RP USH2AKO rabbits. Figure 1 presents 2D OCT images obtained at different time points along the scanning line indicated in Fig. 1f, illustrating the advancement of the syndrome. The control wildtype (WT) image (Fig. 1a) displays a normal retinal architecture with distinct layers, including the internal limiting membrane (ILM), retinal vessels (RVs), choroidal vessels (CVs), sclera, ellipsoid zone (EZ), and retinal pigment epithelium (RPE) layers. In contrast, significant disruption to the EZ layer was observed in the USH2A model at 4 months (Fig. 1b–e). The disruption and degeneration of the EZ layer progressed up to 12 months during the monitoring period. Figure 1f–g showcases *en face* 3D volumetric OCT images, where the entire optic nerve, RVs and EZ degeneration are clearly visualized with high resolution and contrast. Additionally, Fig. 1h represents a selective single orthogonal image from among the 512 B-scan images to provide a 3D volumetric representation. The EZ degeneration area appears as a region of strong OCT scattering when compared to the adjacent tissues. The *en face* and volumetric OCT images provided detailed information about the retinal architecture, facilitating the identification and analysis of the EZ degeneration areas in comparison to surrounding tissues.

Through segmentation of the images to extract the EZ degeneration area, the thickness of the damaged EZ region was measured using ImageJ software (Fig. 1i). At 4 months, the calculated thickness of the EZ damaged area was approximately 55.87 ± 4.55 µm. By comparing the measurements at different time points, it was observed that the EZ damaged area slightly increased by approximately 32% at 12 months (EZ thickness = 73.77 ± 4.10 µm at 12 month). The EZ damaged area remained almost stable from 6 to 8 months, with a thickness of 57.24 ± 4.10 µm at 6 months compared to 59.40 ± 5.01 µm at 8 months. This information further confirms the ability of OCT to evaluate EZ degeneration in the USH2A syndrome model over the long term.

### In vivo color fundus, fundus autofluorescence, fluorescein angiography, and ICGA imaging of USH2AKO rabbit model

**Figure 2:**
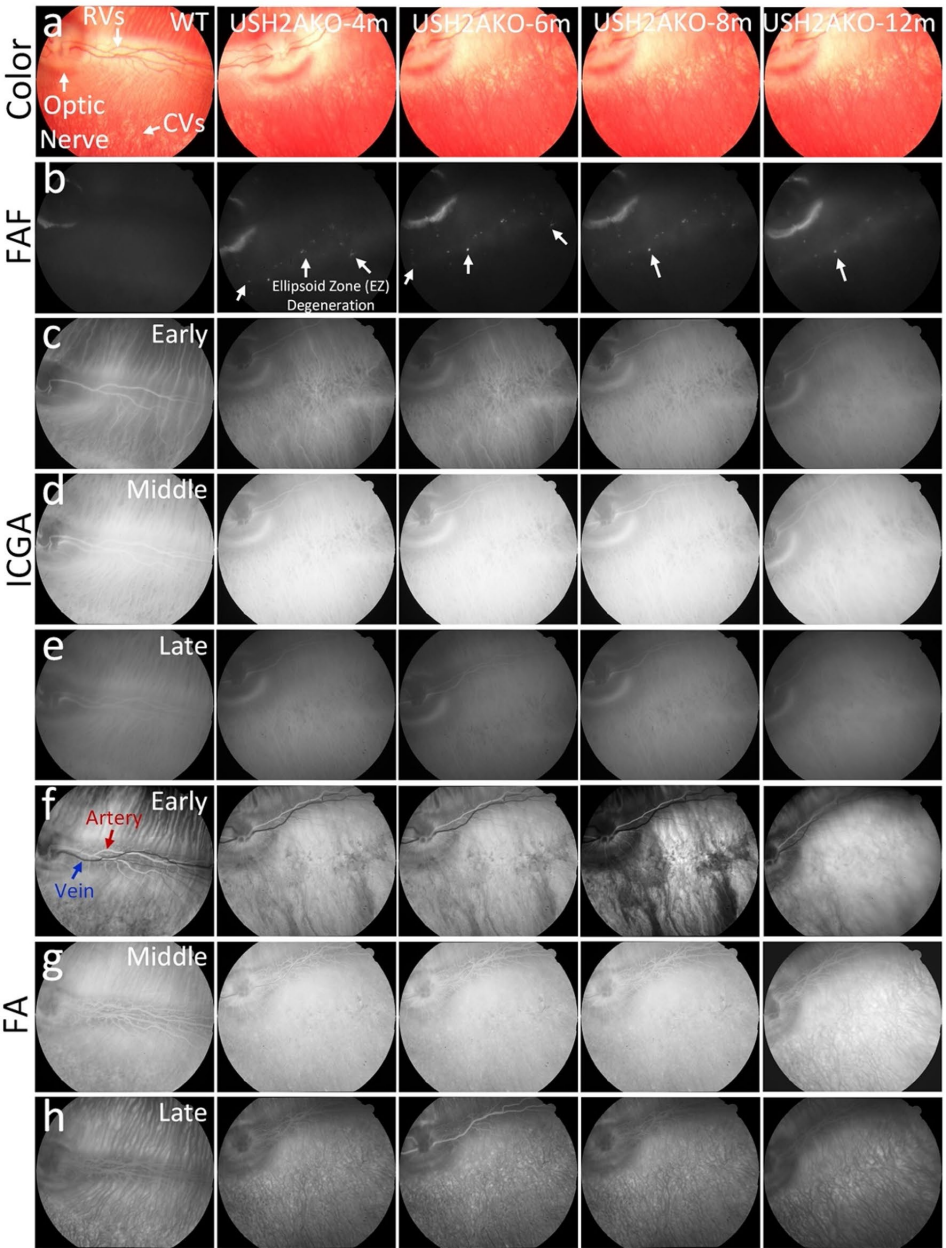
Multimodality imaging evaluation of the USH2AKO rabbit model: (**a**) Color fundus photographs obtained from the control group (WT) showed normal morphology of the optic nerve, retinal vessels (RVs), and choroidal vessels (CVs). Serial images of the USH2AKO group at different time points (4, 6, 8, and 12 months old) also displayed normal morphology of these structures. (**b**) Fundus autofluorescence (FAF) images. In both the WT and USH2AKO groups, a peripapillary hyperFAF crescent was observed, indicating the location of the optic nerve. However, no FAF signal outside this region was detected in the WT group. In contrast, the USH2AKO group exhibited areas of retinal pigment epithelium (RPE) degeneration, which were visible as hyperFAF flecks and regions highlighted with white arrows. These degeneration areas were present at 4 months old and remained observable up to 12 months old. (**c**–**e**) Indocyanine green angiography (ICGA) images obtained at early, middle, and late phases showing the normal morphology of CVs and RVs in both the WT and USH2AKO groups. (**f**–**h**) Serial fluorescein angiography (FA) images acquired at different time points (early, middle, and late phases) did not show any microvasculature changes in either the WT or USH2AKO groups.

Color fundus photography, fluorescein angiography (FA), indocyanine green angiography (ICGA), and fundus autofluorescence (FAF) imaging were performed at different time points (4, 6, 8, and 12 months) on both the USH2A knockout (USH2AKO) rabbit models and wildtype (WT) rabbits, which served as the control group. The color fundus photography images show normal retinal microvasculature (RVs), choroidal vessels (CVs), and the optic nerve in both the USH2AKO and WT groups (Fig. 2a). No evidence of abnormal retinal structure was observed. However, the FAF images obtained from the USH2AKO rabbit models reveal the presence of retinal pigment epithelium (RPE) degeneration (Fig. 2b). These degenerative changes persisted up to 12 months, confirming the stability of the USH2AKO model for longitudinal image monitoring.

To explore the potential of other imaging modalities for visualizing this USH2AKO model, we performed FA and ICGA imaging (Fig. 2c–h). These imaging techniques allowed visualization of the retinal microvasculature, including retinal veins, arteries, capillaries, and the optic nerve, with excellent image quality. Neither FA nor ICGA images showed abnormal structures associated with USH2A syndrome.

### Quantification of the USH2A syndrome marker

**Figure 3:**
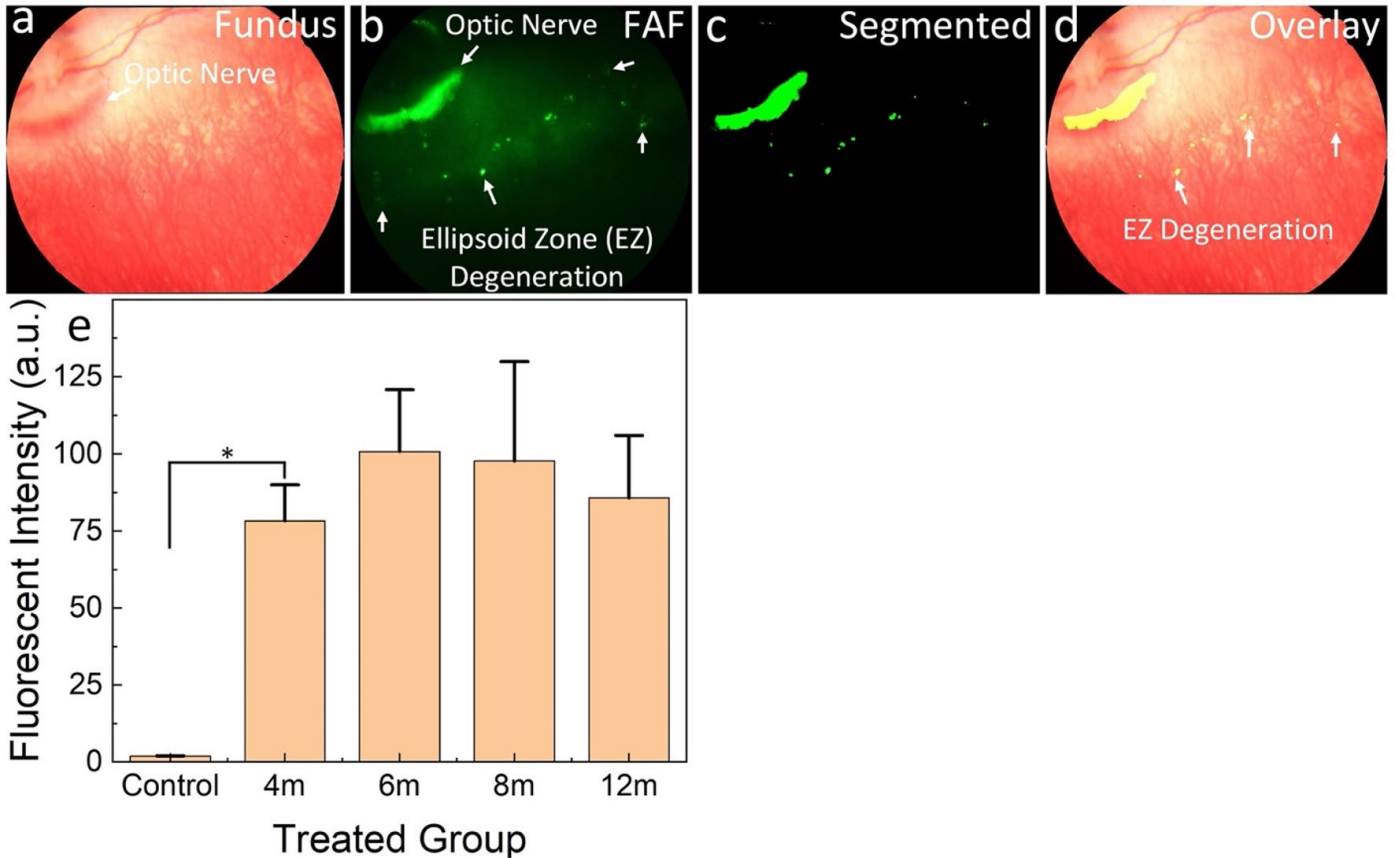
Image segmentation and quantification of USH2AKO rabbit retina at 6 months old: (**a**–**b**) Color fundus and FAF images. (**c**) Segmented images highlighting retinal pigment epithelium (RPE) degeneration area. (**d**) Overlay image combining segmented area with original image. (**e**) Panel of fluorescent intensity. Data presented as mean and standard deviation (n = 3, *p* < 0.005).

To quantify the progression of RPE degeneration over time, we performed image segmentation algorithm to isolate the fluorescent signal detected on the FAF images. The image segmentation algorithm allowed for the precise quantification and analysis of RPE degeneration, providing valuable insights into the progression of the RP in the USH2AKO model. Figure 3b shows a pseudo-green color representation of the FAF image, with enhanced contrast highlighting the location of RPE degeneration. After segmentation, the RPE degeneration areas were clearly distinguished from the surrounding retinal vessels, as depicted in Fig. 3c. By combining the original fundus image (Fig. 3a) with the segmented images (Fig. 3c), the precise locations of the RPE atrophy were visualized, which could aid in improving the diagnosis and monitoring of USH2A syndrome (Fig. 3d).

To further quantify the extent of RPE degeneration, we measured the fluorescent intensity (FI) from the segmented image (Fig. 3c) using ImageJ software. We found that the USH2AKO group exhibited a 54-fold higher fluorescent intensity compared to that of the WT rabbits (Fig. 3e). This fluorescent signal remained relatively stable over time, with a slight fluctuation of approximately 9.5% observed at 12 months compared to 4 months. However, the difference between the FI at 4 months (FI = 85.70 ± 20.20 (a.u.)) and 12 months (FI = 78.28 ± 11.71 (a.u.)) was not statistically significant.

### In vivo PAM imaging of the USH2AKO rabbit model

**Figure 4:**
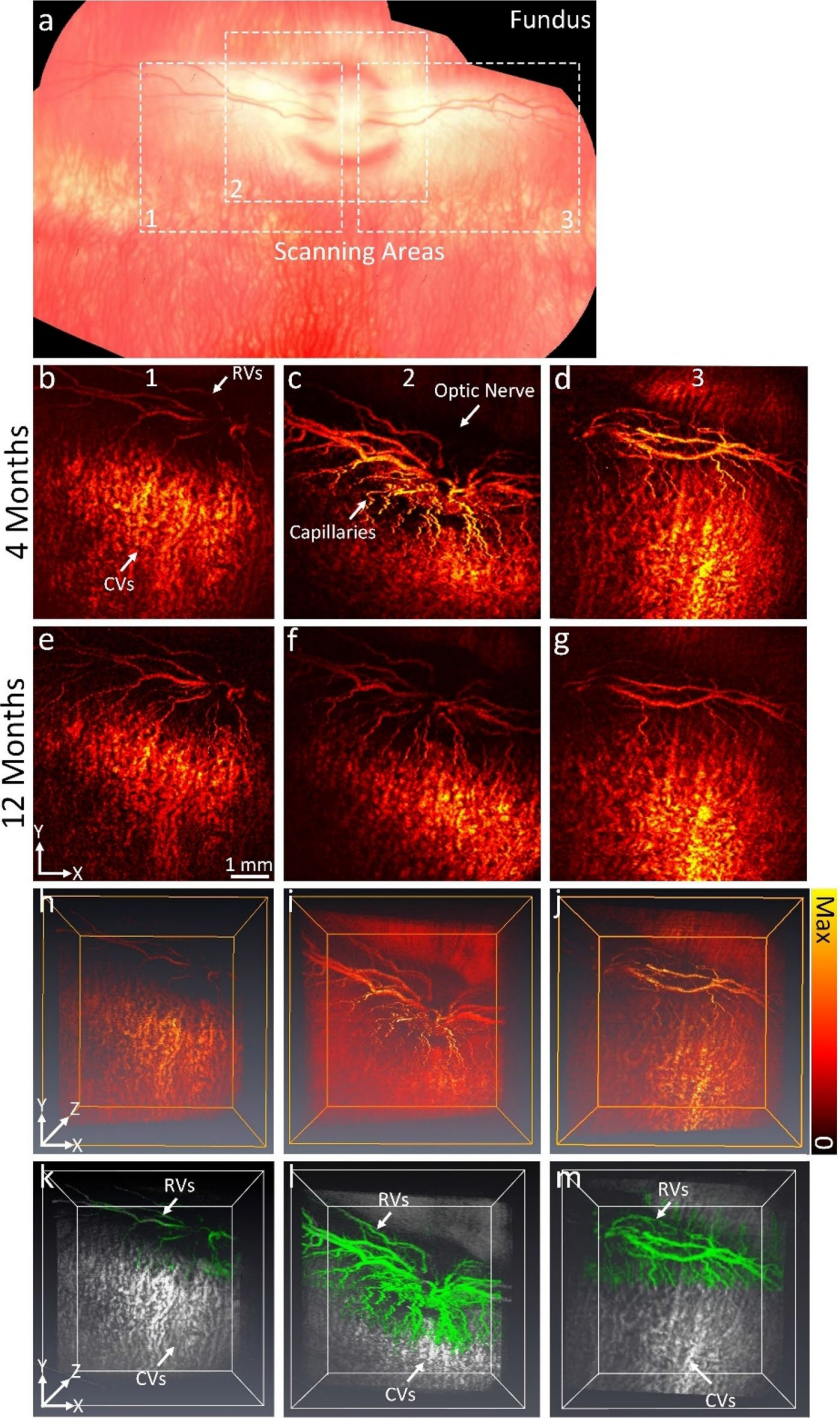
In vivo photoacoustic imaging in USH2AKO rabbit retina: (**a**) Montage color fundus photography showing the entire structure of retina. Inlet numbers indicate the scanning locations. (**a**–**g**) corresponding maximum intensity projection (MIP) PAM images obtained at the excitation wavelength of 578 nm along the scanning areas shown in Figure a at different time points: 4 and 12 months. Retinal vessels (RVs), choroidal vessels (CVs), capillaries and optic nerve were imaged with high resolution and high contrast. (**h**–**j**) 3D rendering PAM images. (**k**–**m**) Segmented 3D PAM images. Pseudo-green color indicates the isolated retinal vessels. Gray color shows the network of choroidal vessels.

To enhance the visualization of the retinal degeneration, photoacoustic microscopy (PAM) imaging was performed on rabbits at 4 and 12 months (Fig. 4). Figure 4a shows a montage color fundus photograph obtained from different areas, including the nasal, temporal, superior, and inferior segments. Corresponding maximum intensity projection (MIP) PAM images along three different scanning areas are illustrated in Fig. 4b–g. These PAM images were acquired using an excitation wavelength of 578 nm, which has strong optical absorption by hemoglobin. The PAM images clearly depict retinal vessels (RVs), choroidal vessels (CVs), the optic nerve, and capillaries with high resolution and image contrast. Figures 4h–j present 3D rendering volumetric data, providing a three-dimensional perspective of the retinal structure. Neither the 2D nor the 3D PAM images provided evidence of a USH2A marker or specific features associated with the syndrome. The diameter of the retinal vessels was not significantly different between the 4-month and 12-month USH2AKO rabbits. Measurements revealed a retinal vessel diameter of approximately 329.13 ± 56.73 µm at 4 months and 319.36 ± 68.38 µm at 12 months. Figures 4k–m exhibits 3D segmented images. The retinal vessels were distinguished from the surrounding choroidal vessels. This result might improve visualization of the dynamic change of the vessels over time and improve understanding of pathology of USH2A.

## Discussion

The USH2A knockout (USH2AKO) rabbit model has been utilized as an important tool for studying the pathogenesis and progression of USH2A syndrome, a genetic disorder associated with moderate to severe SNHL and RP. This animal model provides valuable insights into the understanding of the disease and the evaluation of potential therapeutic interventions. The characterization of the USH2AKO rabbit model involved a comprehensive evaluation using various imaging modalities. Color fundus photography, fluorescein angiography (FA), fundus autofluorescence (FAF) imaging, indocyanine green angiography (ICGA), spectral domain optical coherence tomography (SD-OCT), and photoacoustic microscopy (PAM) were employed to assess the retinal structure, microvasculature, and RPE and EZ degeneration.

Color fundus photography and FA revealed no significant abnormalities in the retinal microvasculature or structure in the USH2AKO rabbits compared to the wildtype control group. However, FAF imaging identified RPE degeneration in the USH2AKO rabbits, which persisted throughout the observation period of up to 12 months. SD-OCT imaging demonstrated the presence of EZ degeneration, demonstrating a significant reduction in the thickness of the EZ layer in the USH2AKO rabbits compared to the wildtype controls. The EZ degeneration was found to progress over time. Additionally, photoacoustic microscopy (PAM) imaging was used to visualize the retinal structure, including the RVs, CVs, optic nerve, and capillaries. PAM imaging did not provide specific markers or evidence of USH2A syndrome in the USH2AKO rabbits likely due to its imaging of hemoglobin in vasculature, which does not change until very late in the disease course. The USH2AKO rabbit model has proven to be valuable for longitudinal studies and monitoring the progression of USH2A syndrome. The longitudinal RPE and EZ degeneration over time and the presence of retinal atrophy observed in this model closely resemble the pathological characteristics seen in human patients with USH2A syndrome.

The RPE is a critical component of the retinal structure, playing a pivotal role in maintaining the health and function of photoreceptor cells. The identified RPE degeneration in our USH2AKO rabbit model may encompass various changes, including disruptions in RPE cell morphology, alterations in RPE cell function, and potential structural anomalies within the RPE layer itself. Drawing parallels between our findings in the USH2AKO rabbit model and human pathological conditions is an essential avenue of discussion. RPE degeneration is a common feature in several retinal diseases, including age-related macular degeneration (AMD), retinitis pigmentosa (RP), and various forms of Usher syndrome. We hypothesize that the RPE changes observed in our model may reflect a process akin to what occurs in some human retinal diseases. Given that USH2A mutations are linked to Usher syndrome, a genetic disorder characterized by retinal degeneration, it is plausible that our findings hold relevance to understanding the pathophysiological mechanisms at play in the human context. The connection between the RPE degeneration observed in our USH2AKO rabbit model and human pathological conditions can shed light on potential clinical implications. It may open avenues for further research into therapeutic interventions and diagnostic markers that target RPE-related degenerative processes, benefiting patients with Usher syndrome and related retinal degenerative disorders.

Another advantange of the multimodal imaging system is that 3D PAM provided high-resolution images with enhanced contrast, allowing for visualization of retinal vessels, choroidal vessels, and capillaries. OCT imaging played a crucial role in visualizing the progression of ellipsoid zone (EZ) degeneration, a hallmark feature of USH2A syndrome. The measurements of EZ thickness over time using OCT provided quantitative data on the progression of degeneration and the degenerative changes observed in the USH2AKO model over the long term. This study highlights the potential of using multimodal imaging for non-invasive and longitudinal monitoring of USH2A syndrome in an animal model. The use of these techniques allows for the assessment of disease progression, evaluation of potential therapeutic interventions, and may aid in the development of novel diagnostic and treatment strategies for USH2A syndrome.

Tsz Kin Ng et al*.* conducted a groundbreaking study that utilized optical coherence tomography (OCT) and autofluorescence for imaging USH2A-associated retinal changes^49^. They reported significant reduction in retinal thickness and extensive disruption in the ellipsoid zone as biomarkers of disease progression. This study collectively underscores the importance of multimodal imaging in elucidating the biology of USH2A-related retinal degeneration. The combined imaging approaches have allowed for the identification of specific markers associated with USH2A, aiding in the early diagnosis and monitoring of the syndrome. In addition, the integration of PAM with other techniques can provide insights into the mechanisms underlying USH2A progression. This study has demonstrated how multimodal PAM can reveal microvascular alterations associated with USH2A, contributing to a comprehensive understanding of the disease.

Furthermore, these multimodal imaging studies have far-reaching clinical implications, such as the potential to improve early diagnosis. By identifying disease-specific markers, clinicians may be able to diagnose USH2A-related retinal conditions at an earlier stage. Understanding the biological mechanism and disease progression can inform the development of targeted therapies for USH2A patients and serve as novel biomarkers and endpoints for the development of new therapeutics. Lastly, multimodal imaging provides a valuable tool for monitoring disease progression over time, allowing for timely interventions and personalized treatment plans. These studies not only enhance our understanding of the biology of USH2A but also hold promise for improved diagnosis and treatment strategies for affected individuals. Our research builds upon these foundational studies to further advance our knowledge of USH2A-associated retinal degeneration.

While our PAM imaging system delivers comprehensive anatomical information on the USH2AKO model, offering high-resolution visualization of retinal vessels, choroidal vessels, and capillaries in both 2D and 3D, it falls short in providing significant insights into the progression of Usher syndrome. This limitation can be attributed to the absence of melanin in the White New Zealand rabbits employed in this study. The absence of melanin in the RPE cells serves as a crucial diagnostic hallmark for RPE degeneration, resulting in a reduction in intrinsic photoacoustic signals. To gain a more profound understanding of the progression of USH2A syndrome, one can consider the use of pigmented rabbits.

In conclusion, this study demonstrates the utility of multimodal photoacoustic microscopy (PAM), optical coherence tomography (OCT), and fluorescent imaging as promising techniques for in vivo monitoring of a reproducible USH2A knockout (USH2AKO) model. The multimodal imaging modalities allowed for the evaluation of the retinal structure, microvasculature, and degenerative changes associated with USH2A syndrome. Thus, this study provides valuable tools for the characterization and monitoring of USH2A syndrome, contributing to a better understanding of the disease and potentially leading to improved management of affected individuals in the future.

## Materials and methods

### Animal model preparation

All animal studies conducted in this research adhered to the ARRIVE (Animal Research: Reporting of In Vivo Experiments) guidelines and followed the ARVO (The Association for Research in Vision and Ophthalmology) Statement for the Use of Laboratory Animals in Ophthalmic and Vision Research. The experimental procedures were performed in compliance with the applicable guidelines, regulations, and ethical considerations. Prior to the initiation of the study, the research protocol (Protocol PRO00010388) was reviewed and approved by the Institutional Animal Care & Use Committee (IACUC) of the University of Michigan.

In this study, six New Zealand white rabbits, including both males and females, aged between 4 and 12 months, and weighing 2.5–3.4 kg, were bred at the Center for Advanced Models and Translational Sciences and Therapeutics (CAMTraST) at the University of Michigan Medical School. The generation and characterization of the USH2AKO rabbits was reported previously^15^. Rabbits were selected in this study due to their similarities to human eyes and suitability for ophthalmic research. Rabbits were housed in a controlled environment with an air-conditioned room. The lighting conditions followed a 12-h light–dark cycle, providing a standard diurnal rhythm. They were given unrestricted access to water and provided with standard laboratory food to meet their nutritional requirements.

Prior to any procedures or imaging, all rabbits were anesthetized using intramuscular injections. Ketamine (40 mg/kg, 100 mg/mL) obtained from JHP Pharmaceuticals (Rochester, MI, USA) and xylazine (5 mg/kg, 100 mg/mL) from Anased® (Boise, ID, USA) were used for anesthesia induction. To facilitate ophthalmic examinations, pupillary dilation was achieved by administering tropicamide 1% ophthalmic and phenylephrine hydrochloride 2.5% ophthalmic solutions. Topical anesthesia was also applied using 0.5% tetracaine or proparacaine to ensure the comfort of the rabbits during the procedures. To maintain corneal hydration throughout the experiments, phosphate-buffered saline (BRL, Life Technologies; Grand Island, NY, USA) was administered regularly, typically every minute. The vital signs of the rabbits, including mucous membrane color, temperature, heart rate, respiratory rate, and oxygen saturation, were continuously monitored before, during, and after anesthesia. A pulse oximeter (V8400D Capnograph & SpO2 Digital Pulse Oximetry, Smiths Medical, MN, USA) was employed to assess oxygen saturation and monitor the respiratory status of the rabbits.

### Multimodal imaging equipment

**Figure 5:**
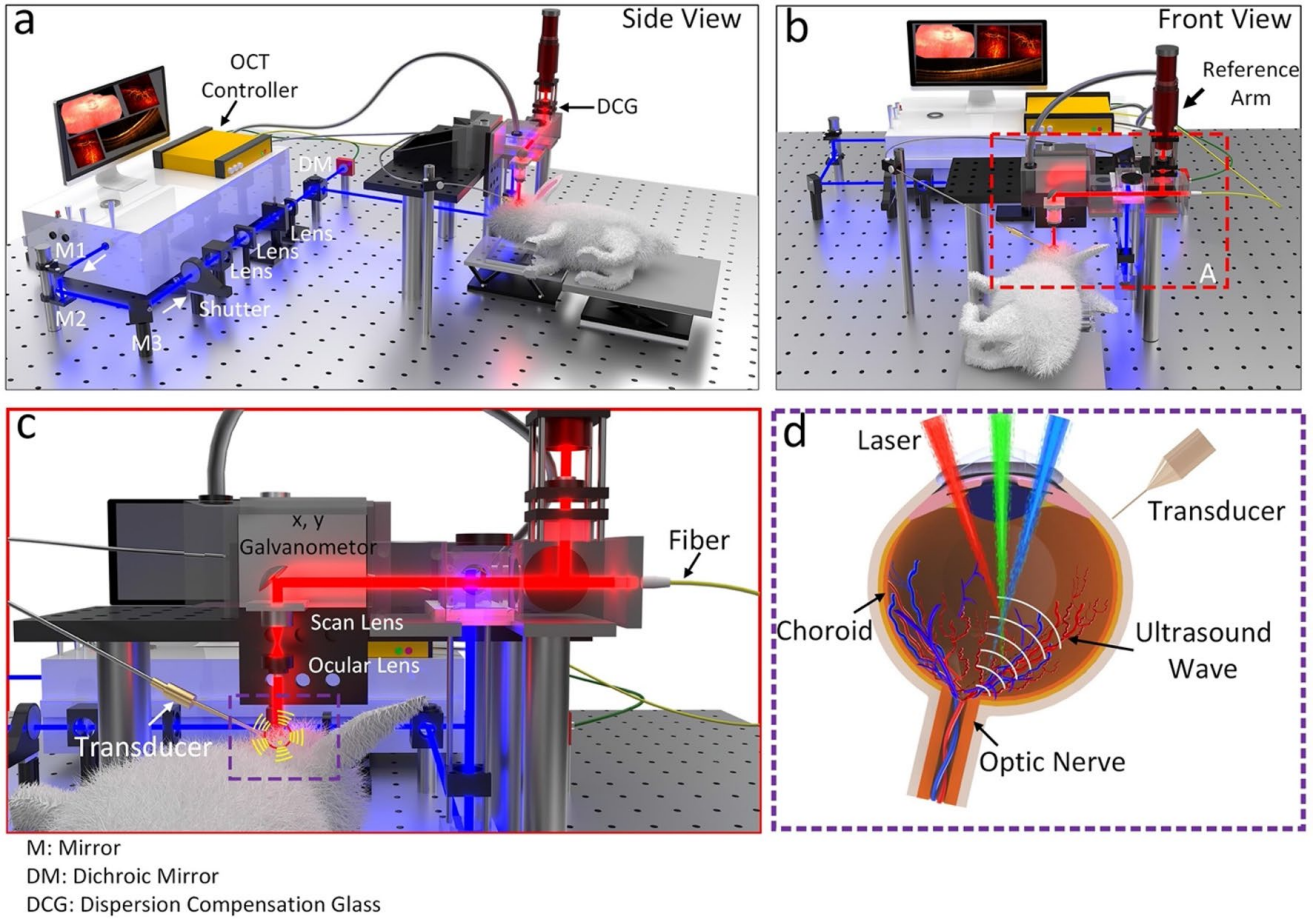
Schematic of the experimental setup multimodal photoacoustic microscopy and optical coherence tomography. (**a**–**b**) Side and front views of the imaging system. (**c**) Magnification of the selected area shown in (**b**). (**d**) Magnification of the violet area shown in (**c**), demonstrating schematic of the retina, position of the transducer in contact with conjunctiva and laser beam focus in the retina.

Multimodality imaging combining SD-OCT and PAM allowed for simultaneous or sequential acquisition of structural and functional information with high resolution and depth of penetration. Figure 5 depicts our multimodal imaging system that combines spectral-domain optical coherence tomography (SD-OCT) and photoacoustic microscopy (PAM)^39,50^. The system, as described by Tian et al*.*, integrates the capabilities of both modalities to provide complementary imaging information^36^. The SD-OCT component of the system was modified from a commercially available system (Ganymede-II-HR, Thorlabs, Newton, NJ, USA). The system included an additional ocular lens following a scan lens, and a dispersion compensation glass (DCG) was added to the OCT reference arm. For the PAM component, an optical parametric oscillator (OPO) (NT-242, Ekspla Vilnius, Lithuania) served as the illumination source. The OPO offered tunability in wavelength (405–2600 nm) and had a pulse duration of 3–6 ns. The PA signal, generated by endogenous chromophores such as hemoglobin and melanin, was detected by a custom-built needle-shaped ultrasonic transducer (Optosonic Inc, Arcadia, CA, USA) with a center frequency of 27.0 MHz. The transducer was in direct contact with phosphate-buffered saline (PBS) on the rabbit conjunctiva. The PA signal was digitized using a high-speed digitizer (PX1500-4, Signatec Inc., Newport Beach, CA, USA) with a sampling rate of 200 MS/s and amplified using a low-noise amplifier (AU-1647, L3 Narda-MITEQ, NY, USA) with a gain of 57 dB.

The center laser wavelength for the OCT system was 905 nm, using two superluminescent light-emitting diodes with center wavelengths of 845 nm and 932 nm. The approximate bandwidth estimation is around 220 nm. The imaging depth of the OCT system was 1.9 mm. The aerial lateral resolution of the PAM system was demonstrated to be 4.1 μm, while the SD-OCT system achieved an aerial lateral resolution of 3.8 μm^51^. The PAM and OCT systems have quantified axial resolutions of 37.0 μm and 4.0 μm, respectively. The laser energy was maintained at approximately half of the ANSI safety limit, measuring around 80 nJ.

### In vivo fundus photography, fundus autofluorescence (FAF), fluorescein angiography (FA), and indocyanine green angiography (ICGA) imaging

All rabbits, including both wild-type (WT) and USH2AKO rabbits, underwent imaging using various techniques, including fundus photography, fundus autofluorescence (FAF), fluorescein angiography (FA), indocyanine green angiography (ICGA), photoacoustic microscopy (PAM), and optical coherence tomography (OCT). The experiments were first implemented with color fundus imaging, followed by sequential imaging using fundus autofluorescence (FAF), fluorescent angiography (FA), and indocyanine green (ICGA). Subsequently, following ICGA imaging, the animal was transitioned to the multimodal PAM and OCT imaging system. The imaging was performed longitudinally to assess progressive retinal structure, vascular perfusion, neovascularization, and the presence of RPE and EZ degeneration lesions. To facilitate the imaging procedures, a pediatric Barraquer wire speculum was placed to keep the eyelids open. Fundus photography was acquired using the Topcon 50 EX system (TRC 50EX, Topcon Corporation, Tokyo, Japan), allowing for visualization of the posterior segment of the eye.

For FA imaging, a 0.2 mL injection of fluorescein sodium solution (Akorn, Lake Forest, IL, USA) was administered intravenously through the marginal ear vein of the anesthetized rabbit. The FA images were acquired immediately after the injection of fluorescein sodium solution followed by every minute, for a duration of 5 to 15 min post-injection. This imaging protocol enabled the monitoring of fluorescence intensity and the detection of vascular changes.

Similarly, ICGA imaging was performed by injecting 0.2 mL of indocyanine green (ICG) dye (HUB Pharmaceuticals LLC, Patheon, Italy). The ICGA contrast was imaged at the same intervals as the FA imaging to assess vascular perfusion and potential abnormalities.

### Fluorescent and OCT signal intensity measurement

To analyze the fluorescence intensity and surface area changes over time, the acquired images were processed using ImageJ software. Image segmentations were implemented to isolate the location of EZ degeneration (Fig. S1). Regions of interest (ROI) were selected at the location of the segmented EZ areas, ensuring the entire margin of the EZ degeneration lesion was included. The average fluorescence intensity and surface area of these regions were determined by ImageJ. To account for background intensity noise, the average of several images from different time points was calculated and subtracted from the values obtained from the ROI. Hyperfluorescent EZ degeneration lesions were identified using the free-hand application in ImageJ, allowing for the delineation of the affected areas. For WT group, a dozen areas were selected at different location on the FAF images (Fig. S2) which is matched with the USH2AKO group. Average signals were determined using ImageJ. By employing these imaging and analysis techniques, the study aimed to evaluate and quantify the characteristics and changes in RPE and EZ degeneration in the USH2AKO rabbits compared to WT rabbits.

### In vivo multimodal PAM and OCT imaging for CNV

To minimize motion artifacts during imaging, an anesthetized rabbit's head and body were positioned on two stabilization platforms. This setup ensured stability and reduced movement during the imaging process. Additionally, a water-circulation blanket (TP-700, Stryker Corporation, Kalamazoo, MI) was used to maintain an appropriate body temperature. An integrated charge-coupled device (CCD) camera provided real-time visualization, allowing researchers to precisely target the region of interest (ROI) during in vivo experiments. This visualization assisted in positioning the imaging probes accurately. The reference arm of the OCT system was calibrated to enhance image quality, ensuring accurate depth information during image acquisition. For the OCT imaging, B-scan images were acquired with a resolution of 512 × 1024 A-lines and an acquisition rate of 36 kHz. The OCT system enabled cross-sectional imaging of the retina, providing high-resolution structural information. To perform multimodality imaging, the PAM system was integrated with the OCT system. The PAM system was controlled using Matlab2019b software (MathWorks, MA, USA). PAM utilized an ultrasonic transducer in combination with optical scanning galvanometers to obtain three-dimensional volumetric PAM images. These images were acquired through raster scanning, capturing the photoacoustic signals from the tissue. The acquired PAM images were further processed and rendered using Amira software. The integration of PAM and OCT into a single system allowed for complementary information from the imaging modalities.

### Statistical methods

The experimental conditions were repeated a minimum of three times, with measurements of fluorescent intensity and OCT signals taken for each condition. An ANOVA analysis was conducted to assess any notable distinctions among the treatment groups. The resulting data was represented as the mean ± standard deviation (SD), with p-values less than 0.05 indicating statistical significance.

### Supplementary Information

Supplementary Figures.

## Data Availability

The data that support the plots and other findings of this study are available from the corresponding authors upon reasonable request.
