# Supplementary material for: Multimodal photoacoustic microscopy, optical coherence tomography, and fluorescence imaging of USH2A knockout rabbits
Source: Sci Rep. 2023 Dec 12;13:22071. doi: 10.1038/s41598-023-48872-1 (PMC10716268; doi:10.1038/s41598-023-48872-1)
Supplement: Supplementary file 1 — Supplementary Figures. [file 41598_2023_48872_MOESM1_ESM.docx]

Supplementary Information

Title: Multimodal Photoacoustic Microscopy, Optical Coherence Tomography, and Fluorescence Imaging of USH2A Knockout Rabbits

**Authors:** Van Phuc Nguyen^1^, Justin Hu^1^, Josh Zhe^1^, Eugene Y. Chen^3^, Dongshan Yang^3*^, and Yannis M. Paulus^1,2* ­­­^

**Affiliations:**

^1^Department of Ophthalmology and Visual Sciences, University of Michigan, Ann Arbor, MI 48105, USA

^2^Department of Biomedical Engineering, University of Michigan, Ann Arbor, MI 48105, USA

^3^Center for Advanced Models for Translational Sciences and Therapeutics, University of Michigan, Ann Arbor, MI 48109, USA

^*^Corresponding Authors:

Yannis M. Paulus, M.D., F.A.C.S.

Department of Ophthalmology and Visual Sciences

Department of Biomedical Engineering

University of Michigan

1000 Wall Street

Ann Arbor, MI 48105, USA

Email Address: ypaulus@med.umich.edu

Dongshan Yang, Ph.D.

Center for Advanced Models for Translational Sciences and Therapeutics Department of Internal Medicine

University of Michigan

2800 Plymouth Rd NCRC B26-355S

Ann Arbor, MI 48109-2800, USA

Email Address: doyang@med.umich.edu

**SUPPLEMENTARY FIGURES**


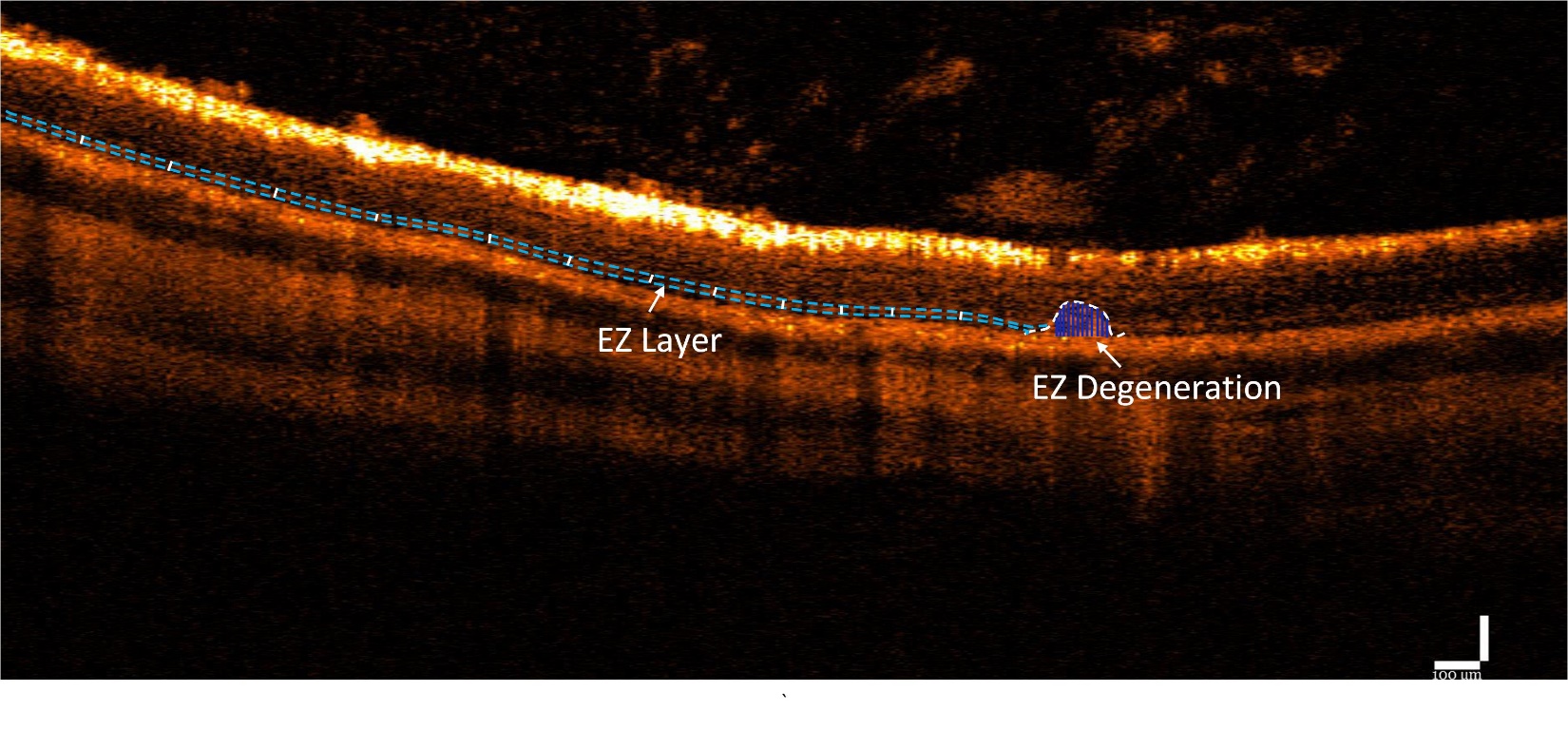


**Figure S1. OCT Signal Intensity measurement.** The margins of EZ degeneration areas were isolated using ImageJ (white dotted lines), while blue dotted indicate the selected region of interests (ROIs) applied to measure the non-degenerated EZ. A total of 12 different B-scan images were selected to quantify the EZ thickness and EZ signal intensity.

**
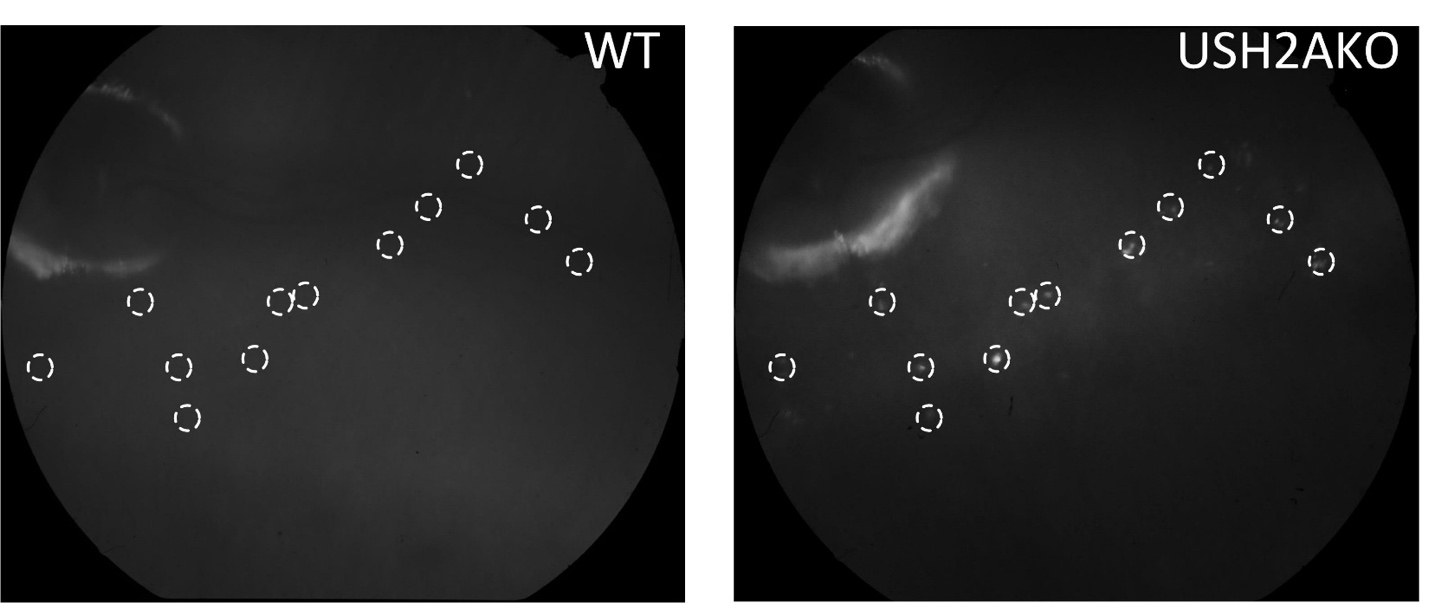
**

**Figure S2. Fluorescent intensity measurement for WT group.** White dotted circles indicate the selected region of interests (ROIs) to measure the fluorescent intensity. These areas were selected at similar location of the EZ degeneration from USH2AKO group.
